# Identification of key genes involved in myocardial infarction

**DOI:** 10.1186/s40001-019-0381-x

**Published:** 2019-07-03

**Authors:** Linlin Qiu, Xueqing Liu

**Affiliations:** Danyang People’s Hospital of Jiangsu Province, Danyang, China

**Keywords:** Myocardial infarction, Incident, Recurrent, Biomarkers, Gene expression differences

## Abstract

**Background:**

This study focuses on the identification of conserved genes involved in myocardial infarction (MI), and then analyzed the differentially expressed genes (DEGs) between the incident and recurrent events to identify MI-recurrent biomarkers.

**Methods:**

Gene expression data of MI peripheral blood were downloaded from GSE97320 and GSE66360 datasets. We identified the common DEGs in these two datasets by functional enrichment analysis and protein–protein interaction (PPI) network analysis. GSE48060 was further analyzed to validate the conserved genes in MI and to compare the DEGs between the incident and recurrent MI.

**Results:**

A total of 477 conserved genes were identified in the comparison between MI and control. Protein–protein interaction (PPI) network showed hub genes, such as *MAPK14*, *STAT3*, and *MAPKAPK2*. Part of those conserved genes was validated in the analysis of GSE48060. The DEGs in the incident and recurrent MI showed significant differences, including *RNASE2* and *A2M*-*AS1* as the potential biomarkers of MI recurrence.

**Conclusions:**

The conserved genes in the pathogenesis of MI were identified, benefit for target therapy. Meanwhile, some specific genes may be used as markers for the prediction of recurrent MI.

**Electronic supplementary material:**

The online version of this article (10.1186/s40001-019-0381-x) contains supplementary material, which is available to authorized users.

## Background

Myocardial infarction (MI) is defined as myocardial cell death due to prolonged ischemia [1]. Worldwide, about 15.9 million MI occurred in 2015. An MI was one of the top five most expensive conditions during inpatient hospitalizations in the US, with a cost of about $11.5 billion for 612,000 hospital stays as estimated in 2011 [2]. The main treatment strategy of MI is myocardial revascularization by the percutaneous coronary intervention (PCI) combined with management of cardiovascular risk factors [3]. Biomarkers are measurable and quantifiable biological parameters which serve as indices for health and physiology assessments. Diagnosis of MI is generally made by combining observation changes in a surface electrocardiogram (ECG) and blood levels of sensitive and specific biomarkers. Overall, the preferred biomarker for each specific category of MI is cTn (I or T) due to its high myocardial tissue specificity as well as high clinical sensitivity [1]. If a cTn assay is not available, the best alternative is MB (muscle/brain) fraction of creatine kinase (CKMB). Elevation of cTn or CKMB in the blood reflects injury leading to necrosis of myocardial cells [1]. In addition, myoglobin, N-terminal proBrain natriuretic peptide, and lactate dehydrogenase have also been considered as clinical diagnosis biomarkers of MI [4]. However, how these biomarkers function myocardial cells injury and necrosis are unclear.

In this study, we identified the conserved genes to investigate the molecular mechanism underlying MI development. Incident MI is defined as the first MI for patients, and it is considered to be a recurrent MI if characteristics of MI occur after 28 days following an incident MI [1, 5]. Differences between first and recurrent events on gene expression profiling are poorly described. Thus, we studied potential differences in the gene expression between patients with an incident and recurrent MI. In addition, little is known of the risk factors of recurrent MI at the transcriptome level. To address this issue, we further detected the potential biomarkers associated with recurrent MI occurrence.

## Methods

### Datasets

We searched the keywords “myocardial infarction”, “peripheral blood”, “GPL570” in the GEO datasets, and obtained 3 GEO datasets-GSE97320, GSE66360 and GSE48060.

GSE97320 and GSE66360 included gene expression profiles of peripheral blood from patients with MI and normal controls. GSE48060 contained gene expression profiling of patients with incident MI and that with recurrent events as well as normal controls. The platform used in these three datasets is GPL570 HG-U133_Plus_2 Affymetrix Human Genome U133 Plus 2.0 Array.

### Differentially expressed gene (DEGs) screen

Gene expression data were first downloaded from each dataset, and the expression levels of genes in each sample were extracted from Series Matrix File(s). And then, R was used to pre-process the downloaded raw data via background correction and quantile normalization. Using Perl [6] probes were transformed into genes. Subsequently, “impute” package [7] was applied to complement the missing expression with its adjacent value.

To screen DEGs between the MI group and the control group, Limma [8] package in R was used. DEGs were screened with |log2(fold change)| > 0.45 and *P* < 0.05.

### Functional enrichment analysis

To obtain the biological function and signaling pathways of conserved genes, GOstats and clusterProfiler [9] packages were used to detect gene ontology categories and KEGG pathways. The threshold of GO function and KEGG pathway of DEGs was all set as *P* < 0.05.

### Protein–protein interaction (PPI) network analysis

To gain insights into the interaction between proteins encoded by DEGs, the database of HPRD [10], BIOGRID [11], and PIP [12] were used to retrieve the predicted interactions of the conserved genes. Then, the PPI network was visualized by the Cytoscape 3.2.1 [13]. A node in the PPI network denotes protein, and the edge denotes the interactions. Cytocluster was further performed to identify the sub-modules.

### Statistical analysis

Data were expressed as mean ± SD. A value of *P* < 0.05 was considered significant.

## Results

### Identification of conserved genes in MI

**Fig. 1:**
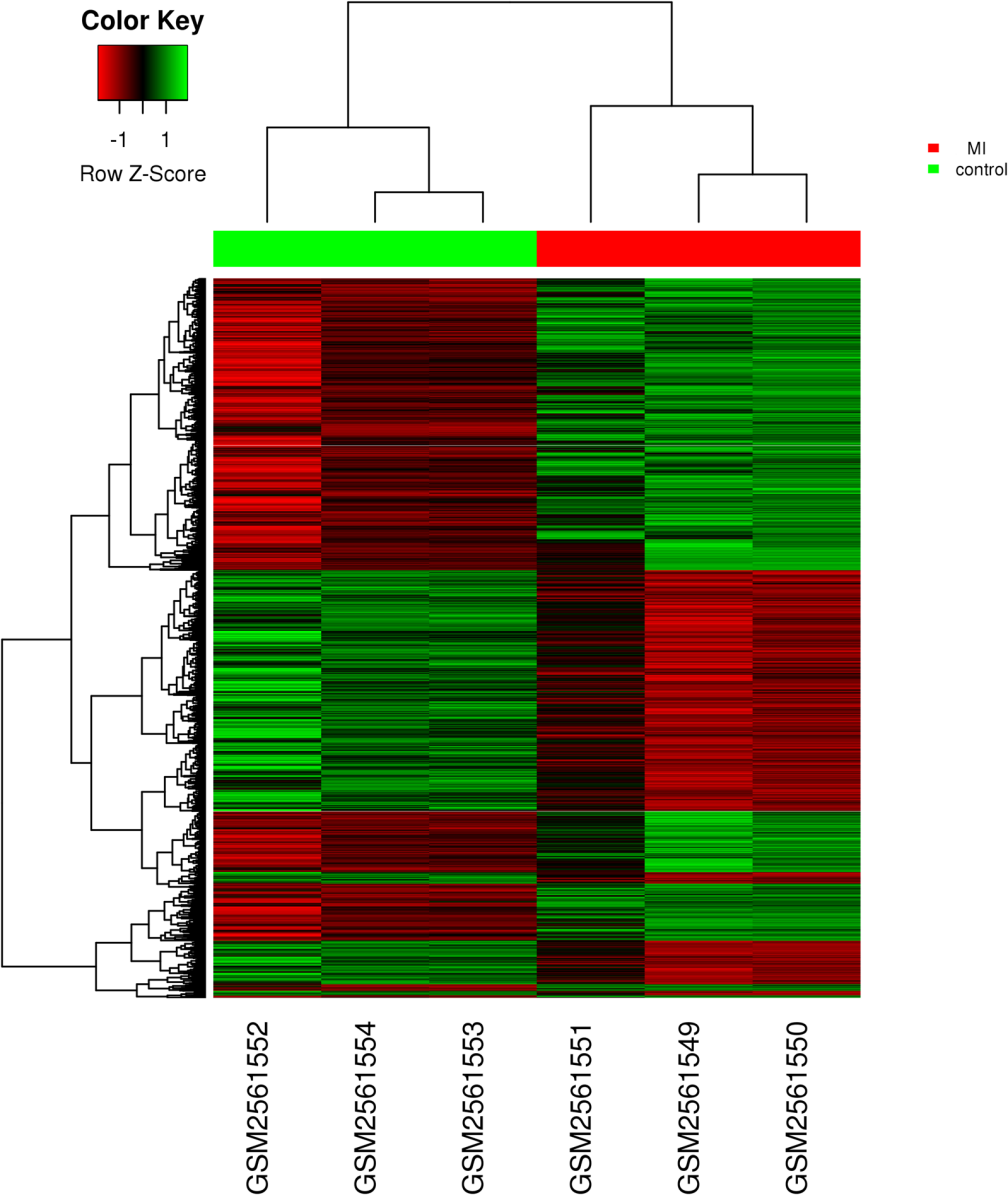
Heat maps for the DEGs in the microarray of the MI patients and healthy controls from dataset GSE97320. The *x*-axis represents the samples and *y*-axis indicates the DEGs

**Fig. 2:**
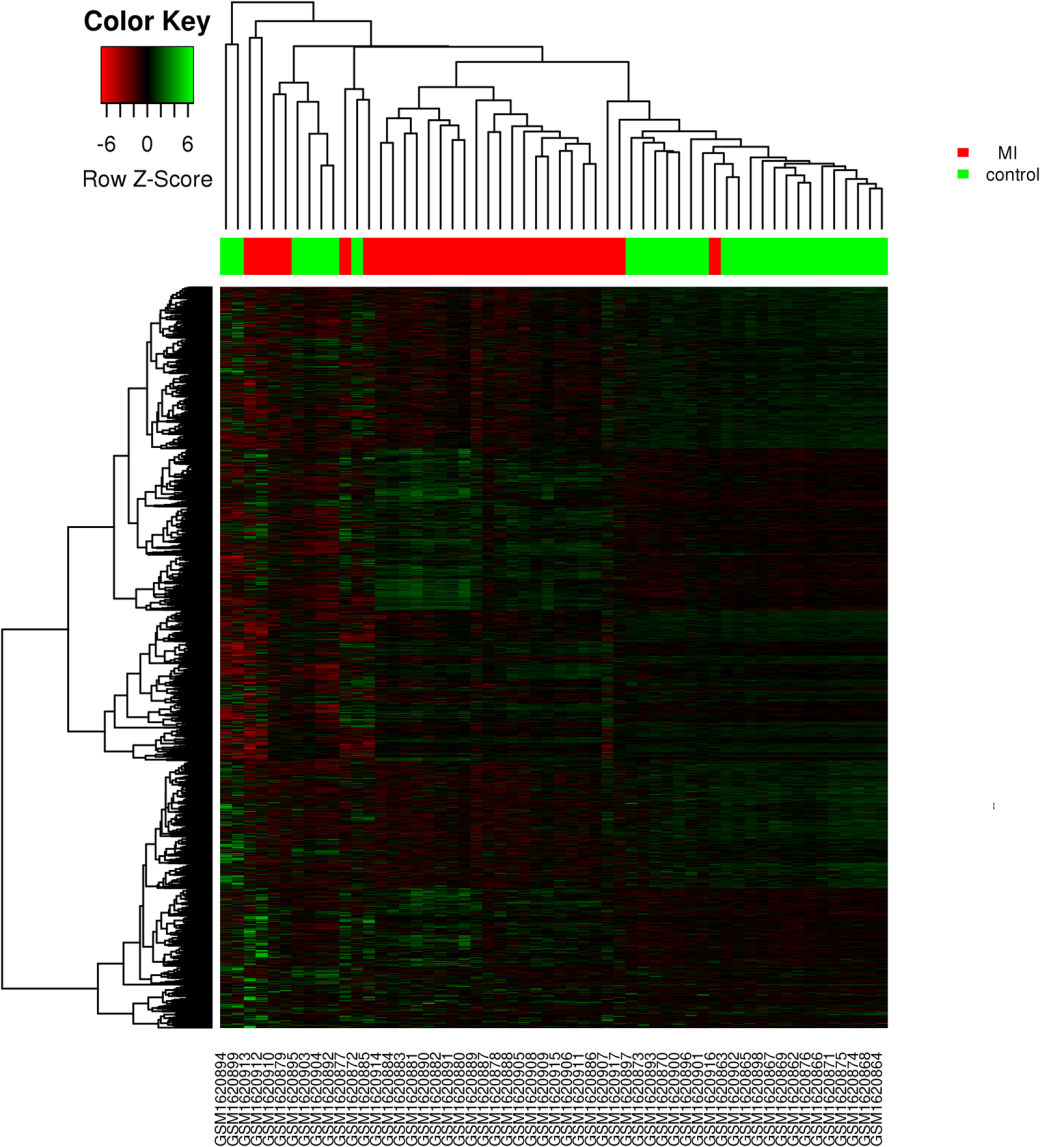
Heat maps for the DEGs in the microarray of the MI patients and healthy controls from dataset GSE66360. The *x*-axis represents the samples and *y*-axis indicates the DEGs

To identify conserved genes involved in MI, comparisons between patients with MI and normal individuals were performed to identify differentially expressed genes (DEGs)in two datasets (GSE97320 and GSE66360), which included gene expression profiles in peripheral blood of patients with MI. A total of 2723 DEGs were identified as the fold change > 1.5 and *P* value < 0.05 in GSE97320, consisting of 1568 upregulated and 1137 downregulated genes (Fig. 1). In GSE66360, 2486 genes including 1141 upregulated genes and 1345 downregulated genes were differentially expressed between patients with MI and healthy individuals (Fig. 2). The genes regulated consistently in GSE97320 and GSE66360 were defined as the conserved genes. A total of 477 conserved genes were differentially expressed in both datasets, including 289 upregulated genes and 188 downregulated genes with the same consistently changed direction (Table 1). These conserved genes may play an important role in the development of MI.

**Table 1:** The conserved genes differentially expressed in both GSE97320 and GSE66360

Conserved genes	GSE97320		GSE66360	
	LogFC	*P* value	LogFC	*P* value
NAMPT	3.715416803	0.000290322	2.319993166	< 0.0001
ACSL1	2.19001047	0.003256902	2.383412763	< 0.0001
S100P	3.760092992	0.004065492	2.468096374	< 0.0001
BCL6	1.877766083	0.001336775	1.757327328	< 0.0001
NFIL3	1.931964423	0.000341343	2.848042441	< 0.0001
ADIPOR1	3.421860496	0.017438642	1.458088512	< 0.0001
MIR8085	0.891511885	0.011998793	1.376148893	< 0.0001
THBD	1.879835689	0.003477466	1.718774941	< 0.0001
IL1R2	3.149278731	0.005306621	2.412986325	< 0.0001
LOC100129518	2.250309635	0.000500285	1.859238466	< 0.0001
C5AR1	2.145806047	0.006632287	2.515427476	< 0.0001
FCN1	0.830570678	0.047479898	1.888141317	< 0.0001
ZFAND5	2.073315077	0.0000438	1.138660839	< 0.0001
IL1RN	1.806072308	0.000219741	1.400798053	< 0.0001
PDE4B	1.306346402	0.01467501	1.270678227	< 0.0001
NFKBIA	1.68182951	0.018260863	1.989220351	< 0.0001
DUSP1	2.764547251	0.000328017	1.271651227	< 0.0001
ZNF137P	− 1.202805686	0.027261213	− 1.682690294	< 0.0001
ITPRIP	1.212871872	0.043664444	1.254857254	< 0.0001
MAPKAPK2	0.765865504	0.038125016	0.778311524	< 0.0001
GADD45A	0.660265341	0.016856452	1.605988515	< 0.0001
BST1	1.232389286	0.029795796	2.187724267	< 0.0001
SERPINA1	3.878586998	0.0000872	1.638136049	< 0.0001
QPCT	2.085892271	0.01667478	2.001432298	< 0.0001
JDP2	1.310855377	0.017420794	1.215915653	< 0.0001
SLC25A37	3.9607872	0.018922169	1.064428591	< 0.0001
GLUL	3.004222492	0.000922447	1.282605825	< 0.0001
S100A9	0.941442135	0.008081851	2.138155827	< 0.0001
HAL	1.388152953	0.003229592	1.15652044	< 0.0001
CLEC7A	2.06910398	0.000914725	1.461369096	< 0.0001
ATP6V0C	1.606678644	0.004965952	1.126718923	< 0.0001
CDA	2.769525669	0.00898907	1.485180438	< 0.0001
TRIB1	2.679234011	0.00045788	1.121985648	< 0.0001
PPIF	1.359533482	0.04082833	1.408576461	< 0.0001
AIF1	1.518215926	0.002104306	1.698436828	< 0.0001
EIF1	1.442005776	0.00017885	0.863524366	< 0.0001
ICAM1	0.729995143	0.019983292	1.394025803	< 0.0001
POLH	− 0.566509776	0.049459364	− 0.737136085	< 0.0001
TREM1	1.502206913	0.019743677	2.603791299	< 0.0001
CCR5	− 0.776384955	0.01829638	− 1.95935621	< 0.0001
PLAUR	0.743728715	0.026751653	1.711115656	< 0.0001
CMTM2	2.866177199	0.00943438	2.053476388	< 0.0001
FOSL2	0.969762074	0.005618694	0.949529005	< 0.0001
LILRA5	1.939542124	0.003227223	1.238799783	< 0.0001
CXCL1	1.597284132	0.013637632	2.179973359	< 0.0001
FCGR2A	3.058858412	0.001506307	1.61933339	< 0.0001
PTAFR	1.254827529	0.021198075	1.110084724	< 0.0001
FCGR2C	1.387185414	0.026898778	0.997833095	< 0.0001
ETS2	1.008783969	0.022408169	1.453545191	< 0.0001
LOC401317	1.325609511	0.01449473	1.356511126	< 0.0001
ZFP3	− 1.181499024	0.028794617	− 1.616668145	< 0.0001
TNFAIP2	1.008366932	0.02131624	1.146286395	< 0.0001
ZNF557	− 0.480686383	0.027928556	− 1.236804343	< 0.0001
IL13RA1	2.154977188	0.007550323	1.297242172	< 0.0001
P2RY13	1.230745211	0.02057251	1.972782405	< 0.0001
SNN	1.528052824	0.001923619	1.00822514	< 0.0001
PADI2	2.10709958	0.000256162	0.906661034	< 0.0001
QKI	0.944951526	0.002191432	0.714960381	< 0.0001
MS4A6A	0.958109791	0.012432822	1.460308619	< 0.0001
LILRA2	1.272631449	0.00994245	1.148478416	< 0.0001
AQP9	2.333240109	0.018284882	2.131747969	< 0.0001
HCAR3	2.824751704	0.00472807	2.110183937	< 0.0001
GRINA	1.402640902	0.028502087	1.074225506	< 0.0001
LOC100128751	− 0.707207366	0.016384142	− 1.043483941	< 0.0001
KDM6B	0.66281941	0.024585326	0.754981016	< 0.0001
GIMAP1	− 0.835067476	0.045419126	− 1.223531516	< 0.0001
BCL2A1	1.943399108	0.021464182	1.895503232	< 0.0001
AMPD2	− 0.721354352	0.048955977	1.614895577	< 0.0001
FPR2	2.199274247	0.02941438	1.600783776	< 0.0001
CPD	0.821859648	0.048035987	1.078872501	< 0.0001
STX11	1.697322241	0.023704466	1.022277137	< 0.0001
TLE3	1.209440706	0.015391784	0.84564545	< 0.0001
GLT1D1	1.606386641	0.007085751	1.505836542	< 0.0001
DGAT2	1.588575564	0.040614788	0.966784955	< 0.0001
SIRPA	1.467435961	0.002424106	0.883668576	< 0.0001
CD93	0.672547012	0.025451375	1.354276298	< 0.0001
PAQR8	− 1.247490335	0.000854948	− 1.105178815	< 0.0001
HERPUD1	1.563752908	0.000217748	0.815993132	< 0.0001
CXCL8	2.613026128	0.003928869	1.549771356	< 0.0001
LOC101929819	0.819452437	0.024240235	0.847572886	< 0.0001
PYGL	1.989921497	0.025319691	1.582056104	< 0.0001
FPR1	2.973441365	0.000645968	1.446062497	< 0.0001
CEBPD	2.386813879	0.000921878	1.23662014	< 0.0001
STAT3	1.780855138	0.011714132	1.024539916	< 0.0001
BTG2	1.596394305	0.001901717	0.889101647	< 0.0001
SLC6A6	2.447756683	0.000438167	0.644662214	< 0.0001
CLEC12A	1.3493504	0.007738217	1.042625117	< 0.0001
SOCS1	0.674899289	0.031132314	0.803066827	< 0.0001
HOTS	0.47497249	0.043443589	1.235734387	< 0.0001
ZNF786	− 0.846155897	0.006163145	− 1.330967712	< 0.0001
KDELC2	− 1.126543214	0.013735129	− 0.871863341	< 0.0001
SEC14L1	3.222121	0.005523564	0.868185585	< 0.0001
CHI3L1	2.697015762	0.017524244	1.18798994	< 0.0001
RNASE2	0.907871679	0.001791958	1.849318307	< 0.0001
MPP1	1.44935076	0.039373731	1.457675515	< 0.0001
PQLC1	1.098874935	0.039814237	0.508070689	< 0.0001
TCEB3-AS1	− 0.694870463	0.04616438	− 1.433351491	< 0.0001
TIGD7	− 0.985252239	0.036510489	− 1.167389496	< 0.0001
PGD	1.901669081	0.000154316	0.565073179	< 0.0001
U2AF1	1.702264526	0.018461448	0.915025557	< 0.0001
AKIRIN2	1.785779636	0.00034891	0.657209785	< 0.0001
LBH	− 0.91504058	0.032471404	− 1.324336977	< 0.0001
RAD54B	− 0.538426791	0.036659702	− 1.414883543	< 0.0001
MME	2.276317524	0.047321026	1.137294855	< 0.0001
DOCK5	1.078051413	0.032816176	0.835988452	< 0.0001
ABHD5	0.735179941	0.03520765	0.727680923	0.000101662
PLBD1	0.969302605	0.032765571	1.798031132	0.000102992
BACH1	1.351200785	0.00167848	0.585528821	0.000107046
ZYX	1.085019322	0.010168639	0.715711002	0.000108507
FCGRT	0.70475403	0.01525679	0.91961625	0.000111858
GEMIN5	− 1.47500487	0.046026587	− 1.57933263	0.000112299
LOC221272	− 1.181714286	0.010168237	− 0.87301352	0.000115697
TNFRSF10C	1.988784507	0.016690641	0.687475626	0.000115702
TLR4	1.28311422	0.009728893	0.796509712	0.000120788
CDV3	1.709600858	0.0003786	0.756754733	0.000121731
USB1	1.012045755	0.00547019	0.525968344	0.000127289
MXD1	3.02456454	0.003699774	1.099434156	0.000129507
VNN2	2.180194614	0.0109879	1.397451606	0.00013196
SGK223	− 1.252002755	0.000726559	− 0.787841536	0.000138471
TET1	− 0.703607148	0.008657374	− 0.818610782	0.000149306
LPCAT2	0.85182211	0.047333209	0.936460647	0.000150743
MGAM	3.213431913	0.005317158	1.610096737	0.000154249
NPL	1.239490617	0.006173363	0.732825202	0.000169972
LY96	1.346013773	0.005411258	1.029764841	0.000170653
PTGS1	0.967130969	0.003641179	0.77481882	0.000179849
SLC2A14	1.291691746	0.017168802	1.307076894	0.000180583
GIN1	− 1.237598849	0.038502248	− 1.525016605	0.000185774
TKT	1.773187189	0.000256431	0.833485867	0.000189348
CSF2RB	2.200868771	0.008362239	1.631201272	0.000194977
MMP25	1.898237252	0.010803144	0.694482086	0.000198083
CNOT6L	1.556044518	0.003364024	0.596626207	0.000203962
TP53INP1	1.731770541	0.002114113	0.512674471	0.000205394
CLP1	0.660538961	0.039295856	0.702552153	0.000224265
FAM198B	1.263612775	0.018980734	1.063744226	0.000232443
ZNF606	− 1.021874256	0.010996345	− 0.986409189	0.000239356
PECAM1	1.133988939	0.002525393	0.812308902	0.000251335
CPEB2	0.509987239	0.029154597	0.733979649	0.000260216
SHQ1	− 0.690969237	0.034617547	− 1.158028865	0.000264123
FCGR3B	3.813483966	0.008808688	1.227532333	0.000268305
SIAH1	1.165012649	0.015051532	0.902135148	0.000293066
FCGR1B	1.510242008	0.044692504	1.262470054	0.000312948
ZNF30	− 1.110993329	0.02752944	− 0.993962406	0.00034754
MRPS17	− 0.696898862	0.030343011	− 0.929231267	0.00036119
EIF2B3	− 0.522846432	0.034922941	− 0.859607844	0.000415198
ZNF260	− 1.283202812	0.04751078	− 1.223608639	0.000416573
ZBTB3	− 0.838180298	0.021768338	− 1.025353413	0.000421081
SPI1	1.242116323	0.025822193	0.852205923	0.000421285
ELP4	− 0.985373014	0.031611917	− 1.13057002	0.000441549
STK17B	1.634772394	0.016110753	1.070768411	0.000443755
CXCL16	1.430929047	0.019642805	1.517379129	0.000445953
CYP4F3	2.891652429	0.012098196	1.083712332	0.000464153
ZCCHC17	− 0.527154589	0.03711239	− 0.703895685	0.000466123
BNIP3L	2.939399055	0.036736625	0.868766712	0.000480117
HLTF	− 2.085425559	0.00830834	− 0.92642157	0.000481112
ZNF280B	− 0.889975527	0.007382474	− 0.591810379	0.000482869
ENTPD1	0.833331837	0.013979206	0.872451565	0.00048829
SMARCAD1	− 1.536179035	0.00212696	− 1.388394318	0.000503169
ZDHHC18	0.970662172	0.03919739	0.464432831	0.000516733
SP3	0.566964411	0.027129826	0.45352211	0.000530533
DENND1C	− 0.994967054	0.005926672	− 0.720422053	0.000556354
ARHGEF40	0.677302641	0.00863392	0.949974076	0.000560322
VNN3	1.459070601	0.002685799	0.818145005	0.000567591
PTPN12	1.900952269	0.006990223	0.648074395	0.000590111
IL22	0.785725374	0.033763579	0.870706647	0.000608321
AKAP13	1.15374636	0.024380781	0.457987789	0.000610161
HIPK1	0.790715098	0.028493908	0.594190441	0.000620025
SLC2A3	1.277853147	0.005001915	0.975255592	0.000625103
MRPL18	− 0.962891471	0.001294926	− 0.810866571	0.000673172
PNRC1	0.84100527	0.038963808	0.771737601	0.000685328
SRPRB	− 0.572880851	0.02337171	− 0.794559375	0.000695958
IFNGR1	1.303124037	0.013360086	1.017612578	0.000703371
NIF3L1	− 0.869517958	0.043390346	− 1.019489489	0.00070353
XPA	− 0.90413844	0.041510611	− 0.80325959	0.000756448
MMP9	2.441007592	0.01581582	0.97531267	0.000777516
NCF1C	0.855445347	0.038108625	0.783883625	0.000784817
DET1	− 0.81533148	0.005926379	− 0.854080807	0.000803216
COQ10A	− 0.933289637	0.006719525	− 1.081765186	0.000832872
UBALD2	0.844767801	0.034723888	0.610479487	0.00088448
JMJD1C	1.363565695	0.045081671	0.806155508	0.000897176
GNB4	1.332152673	0.000181302	0.703062789	0.00092457
SIRPB1	1.376479024	0.015121968	0.783168056	0.000935223
TBXAS1	0.901392581	0.035582908	0.72491305	0.000938466
LOC100996286	− 1.15942957	0.001304937	− 1.10912258	0.000951423
FUNDC1	− 1.016071403	0.025599739	− 1.17719563	0.000962182
CDC42	1.293690918	0.001559527	0.617592666	0.000979709
CHMP4B	1.337085004	0.024622123	0.789355603	0.001035706
MIDN	1.798309942	0.003199858	0.549277047	0.001044485
ZNF232	− 0.974081458	0.026063953	− 1.280741756	0.001082083
S100A8	1.151105372	0.000612836	1.667435842	0.00114641
SIGLEC5	1.528578408	0.022982592	1.145057625	0.001148462
RAE1	0.786226017	0.04583837	0.646827746	0.001155134
FMNL3	− 0.911262187	0.006347222	− 0.708906078	0.001168614
FFAR2	3.054926626	0.007540706	1.110136702	0.001188049
KYNU	0.913892454	0.003039259	0.770873995	0.001243107
ASAH1	1.098421547	0.005150015	0.754043428	0.00125101
PLAC8	− 0.839086258	0.031308986	− 0.849482287	0.001277728
LINC00909	− 1.345332297	0.013596272	− 0.969000288	0.001316741
PTGS2	2.350439432	0.001568506	1.426523404	0.001319682
PIGF	− 0.808285785	0.036923826	− 0.594491349	0.001339096
ZNF284	− 2.184588821	0.040088141	− 0.93615243	0.001361012
LOC102724851	− 1.155228734	0.013040415	− 0.518495367	0.001376683
STT3A	− 0.929412935	0.006056463	− 0.913154729	0.001399971
ATG3	2.102229556	0.000178319	0.841794717	0.001410075
TIMM9	− 1.40383474	0.007805487	− 0.735973926	0.001444816
TOMM40L	− 0.779793242	0.021017904	1.086712809	0.001468786
ALPL	1.60229352	0.038415425	0.716082883	0.001490739
DSC2	0.98658481	0.010627222	0.567380206	0.001504916
HLX	1.349039117	0.020807799	0.513207382	0.001520858
LYL1	1.538740411	0.040216887	0.502710942	0.001523024
SESN3	2.636101881	0.004536708	0.494696947	0.00155925
RNF141	1.445983931	0.004029835	0.552945436	0.001562541
GABARAPL3	1.014160119	0.002281996	0.717868982	0.001606081
LOC105376805	− 1.365943289	0.001327507	− 0.595216422	0.001625267
GNAS	3.247649892	0.0000955	0.478498353	0.001735362
PTP4A1	0.778394075	0.009116649	0.636669015	0.001778326
UBASH3A	− 1.430887468	0.002003066	− 0.913772788	0.0018777
HSD17B7	− 1.721065936	0.009279288	− 0.835575098	0.001927602
TP53RK	− 0.505724162	0.044175513	− 0.997261329	0.00197887
SERPING1	0.915484027	0.029789731	0.839308791	0.002037968
DOCK4	0.713528467	0.026268016	0.661229341	0.002047801
RBM4B	− 0.754993386	0.025500124	− 0.973299711	0.002076982
GAS7	0.652023956	0.030611426	0.728288754	0.002160493
RNF10	2.292398494	0.033636936	0.623649579	0.002172112
LINC00623	− 1.165387619	0.00087698	− 0.539399165	0.002174586
YBX3	4.365021825	0.003169993	0.52927351	0.002242851
ERGIC1	1.693282707	0.006494326	0.482795744	0.002365768
MARCKS	1.896513739	0.011966121	1.008557895	0.00237302
FTH1	0.721676035	0.006408239	1.182091335	0.002417955
LMO2	1.861941801	0.004174164	0.544068211	0.002425819
ADM	1.851219504	0.008897467	1.254434919	0.002493206
SCYL3	− 1.011979188	0.035057218	− 0.710888004	0.002503157
ZNF140	− 1.418751894	0.021858458	− 0.821318547	0.002526554
RASSF5	1.494672795	0.000190407	0.666636746	0.002625559
ZNF746	1.43259319	0.038940531	0.546212963	0.002633285
NME6	− 0.610227552	0.041831331	− 0.803569365	0.002640731
TFRC	0.605142119	0.044216485	0.486955837	0.002659029
ASCC2	3.012960368	0.048869583	0.503342235	0.002691189
TCL1A	− 1.418926748	0.043956086	− 0.740787553	0.002719864
ADGRG3	1.756451065	0.026679736	0.924004639	0.002740384
RAB1A	2.053694233	0.000197952	0.451082876	0.002771411
CHST15	1.38976538	0.010674237	0.849343314	0.002942074
TNFAIP6	2.840950381	0.005912694	1.108939843	0.002978094
LOC102724229	− 1.48967097	0.03404748	− 1.014128699	0.003062724
MAPRE1	0.531042713	0.021090503	0.459089464	0.003165946
ABHD2	1.201422325	0.005387136	0.46795845	0.003218277
MNAT1	− 0.83937625	0.024109007	− 0.565184788	0.003355533
TMCC3	1.745352413	0.013173346	0.799248122	0.003376755
POLR1B	− 0.772923097	0.032500554	− 0.505466721	0.003452495
PLEKHG2	0.569547478	0.038813449	0.535246343	0.003585976
RBM47	1.784627427	0.001016962	0.678723979	0.003615633
POLE2	− 0.524011745	0.033009479	− 0.878657539	0.003617168
REPS2	1.718120007	0.003180389	0.644368871	0.003635816
GBP3	− 1.845504585	0.008969037	− 1.193440354	0.003699308
ERVK3-1	− 1.050961105	0.014574394	− 0.80126366	0.003700902
TIMP2	1.321414802	0.006004518	0.738209029	0.003701828
JUND	2.816842846	0.00000849	0.466923422	0.003713308
PTGER4	1.137141559	0.044765741	0.758160268	0.003753251
PHC2	1.794885166	0.00447288	0.579182361	0.003765784
RELL1	1.188903575	0.004557879	0.581780349	0.003843326
PDCD11	− 0.669519886	0.031226669	− 0.585698567	0.00386508
LOC101928291	− 0.607349146	0.02839692	− 0.994929526	0.003919704
DNAJC19	− 0.68231173	0.018596595	− 0.534281446	0.003949704
ITGAM	1.858485959	0.0000309	0.668737696	0.003973806
A2M-AS1	− 1.155442856	0.039842116	− 1.138539809	0.003974972
SMCHD1	1.604667418	0.010852317	0.712810012	0.003983746
ICAM2	− 0.984018887	0.017489313	− 0.88255297	0.004009051
CEBPB	0.598014744	0.031789552	1.317994945	0.004020206
SSFA2	1.066587737	0.002451362	0.624973739	0.004021925
PTPRCAP	− 0.866500312	0.011837444	− 0.726859417	0.004052694
POP1	− 0.925608754	0.030781593	− 0.818205615	0.004246787
BLNK	− 1.398591377	0.030430259	− 0.894692561	0.004288343
GALM	− 0.713557563	0.034952709	− 0.675235843	0.004443763
SEC61B	1.180469063	0.000723465	0.686145648	0.004453778
MAP7D3	− 0.553624737	0.025768455	− 0.507870015	0.004521575
GCA	1.509319178	0.004363002	1.192393745	0.004633368
LGALSL	1.21757196	0.044976788	0.671933026	0.004880392
ARAF	1.427113051	0.038366364	0.51978633	0.004913297
RNF144B	0.726950076	0.012122937	0.779524204	0.00500161
KCNJ15	1.979657868	0.039608194	0.628206301	0.005135068
CD40LG	− 0.792190382	0.044206155	− 1.032646424	0.005145558
CPPED1	1.076942495	0.036499438	0.612705064	0.005168794
RIOK3	2.075563218	0.002003992	0.521802306	0.005218069
DDX46	− 0.919216329	0.029662572	− 0.639785975	0.00523761
CDK17	1.128464094	0.013744211	0.693411752	0.005347246
MIR21	1.91559852	0.046387482	0.544304499	0.005431751
SPIN3	− 0.857422597	0.023843176	− 0.769503059	0.005588091
FAM46C	2.601440153	0.021586082	1.005819825	0.005625886
HIST2H2AA4	2.093741596	0.004304829	0.841019575	0.005688879
LOC101930115	− 0.68275475	0.040474709	− 0.607842314	0.005704428
LOC151657	− 1.607243608	0.001601903	− 0.717618669	0.005720524
CLU	2.316583335	0.001072876	0.563627036	0.005777839
AKTIP	− 0.847566702	0.040054537	− 0.633465882	0.006096883
NINJ1	0.945159729	0.010864115	0.87230313	0.006320781
ZFP30	− 1.038905821	0.010392043	− 0.871623208	0.006325373
EIF1B	3.692853038	0.000758987	0.878422088	0.006414737
LOC101930363	− 2.709073019	0.011781869	− 0.736376913	0.006454992
TANK	1.653093179	0.002146166	0.520972495	0.006474026
PARG	− 1.086961793	0.043658497	− 0.724468917	0.006491873
TEFM	− 1.027675228	0.023860331	− 0.667630909	0.006617711
ASAP1	1.098840052	0.016823868	0.514404458	0.006851203
CDKN2D	1.952060497	0.018541817	0.490974421	0.006891249
TSPYL1	2.709566348	0.001494149	0.60819847	0.006988834
CSTF3	− 1.18690418	0.003957706	− 0.491540334	0.006989296
MROH7-TTC4	− 1.067721766	0.016410638	− 0.919365541	0.007060206
RFX5	− 0.538517443	0.041897931	− 0.645397188	0.007076645
NKG7	− 1.070684122	0.010774919	− 0.945817311	0.007078395
DARS2	− 0.78027652	0.027042938	− 0.741649853	0.007137998
ZNF615	− 0.777419719	0.020695789	− 1.209541334	0.007310157
ADSS	1.070420831	0.006216282	− 0.538713686	0.00738181
OGFRL1	1.62280147	0.0000662	0.745837652	0.007530407
CD2	− 0.908924186	0.049667697	− 0.9371322	0.007535782
DYNLL1	− 0.757353509	0.012942379	− 1.226469494	0.007808299
SEPHS1	− 0.960674944	0.02404333	− 0.491689916	0.007844444
AGFG1	0.697014119	0.040811266	0.599122876	0.007932363
WTAP	1.415043348	0.013255137	0.504140918	0.008157105
RNASEH2A	− 0.567107275	0.048285518	− 0.588491225	0.008261927
LCLAT1	− 1.191926318	0.000330923	− 0.907358841	0.008467512
GNA13	2.465840893	0.000677556	0.803476183	0.008677402
HBD	4.363118946	0.03133988	0.714000351	0.008706877
CA5B	− 0.761733369	0.010733624	− 0.717511936	0.009153528
WDR26	1.795069926	0.006752784	0.554641276	0.009208138
BHLHE40	1.067828924	0.01253442	0.82013057	0.00931423
DCTN4	0.995446804	0.019331887	0.602818796	0.009817669
RARRES3	− 0.810774795	0.042801728	− 0.815285574	0.009897921
MRVI1	1.380868974	0.03671614	0.499460438	0.009923543
SLC7A6OS	− 0.747296638	0.003754511	− 0.683052886	0.010144899
LOC100289090	− 0.510049286	0.048064071	− 0.526694355	0.010151137
WDR1	1.684601866	0.000168948	0.468754984	0.010157612
ANXA2R	− 1.735263881	0.001346797	− 0.842518717	0.010211973
LOC101927929	− 0.911010924	0.036707986	− 1.056783587	0.010586272
DCP2	1.339414838	0.012814635	0.536456476	0.010623367
IL7R	− 1.38550515	0.027408601	− 1.003290446	0.010704747
DPY19L2P2	− 1.343859858	0.006832435	− 0.691688548	0.010708918
LRMP	1.425535165	0.019422696	0.548187496	0.010789867
HPR	0.823889667	0.033804337	0.511405624	0.01081264
CFB	1.123068147	0.049371972	0.457666939	0.010904784
LOC284513	− 0.892129855	0.004236477	− 0.701299207	0.010990889
RAB20	0.821602882	0.035312406	0.586702846	0.011071966
FBXO7	3.643752431	0.018286086	0.499532856	0.011346495
PHAX	− 0.789847996	0.035012859	− 0.532516036	0.011379085
BLVRB	2.656708226	0.011781419	0.642487027	0.01141343
WLS	1.657097425	0.028300616	0.62402017	0.012075619
MUT	− 1.050168894	0.002313509	− 0.626508717	0.012205507
LOC100287896	− 0.86494196	0.035810543	− 0.919630542	0.012517769
HSPC102	0.916580698	0.025392925	0.868444451	0.012523152
TSC22D3	2.405997523	0.0000286	0.488948844	0.012661968
PTENP1	2.094685709	0.000479594	0.618312388	0.012790096
ZNF57	− 1.343945962	0.010152991	− 0.929510368	0.012800761
MUTYH	− 0.936725766	0.008091841	− 0.553024313	0.013128222
HCST	− 0.791970019	0.007490591	− 0.50645902	0.013285635
LOC100507616	− 0.521527416	0.042217852	− 0.465453565	0.01346017
CYBB	2.398594516	0.000400338	0.626735409	0.013536222
TIMMDC1	− 0.747343418	0.014838492	− 0.93807409	0.013541407
KIF13A	0.930338853	0.011402054	0.515997085	0.014285432
C14orf169	− 1.092295613	0.006077514	− 0.461703063	0.014457478
ISCA2	− 0.673334196	0.014793629	− 0.801341203	0.014570854
CR1	0.980739302	0.03282439	0.564267726	0.014685731
SMYD4	− 0.743050454	0.008096331	− 0.756746486	0.014705624
MTURN	1.229572779	0.04943481	0.614334998	0.015126434
FASTKD1	− 1.54680628	0.002039946	− 0.7227908	0.015176612
PIGN	− 0.708552558	0.011836956	− 0.49162495	0.015256966
TESPA1	− 1.021501179	0.048240998	− 0.521959366	0.015269293
HOXB2	− 0.940925367	0.027917932	− 1.044582695	0.015348671
TAF3	− 0.874538712	0.016049188	− 0.520997598	0.015458366
MNDA	1.053933023	0.042861478	0.983216212	0.015528146
CDC42EP4	0.498947026	0.039781999	0.532259022	0.01560414
GPD1L	− 0.761261068	0.038421959	− 0.915958276	0.015789382
BBS10	− 1.031497448	0.038982743	− 0.656567662	0.016094327
OR2A9P	− 0.633389532	0.024730134	− 0.565938071	0.016339377
G6PD	0.871501908	0.006760087	0.459398494	0.016352781
TFG	0.720616794	0.006867605	0.487139927	0.016532991
FAM114A2	− 0.459915629	0.040407886	− 0.57057389	0.016675289
ATP1A1	1.12474105	0.013429616	0.707444754	0.01694022
GDE1	1.401322239	0.030249245	0.540437863	0.017493186
RNF170	− 0.504220106	0.023599634	− 0.490772966	0.017518558
SH3BGR	− 0.857459864	0.028655056	− 0.62930005	0.017522267
LOC283588	− 1.368743502	0.045421947	− 0.7194058	0.018040997
PRKCQ-AS1	− 1.188507039	0.019254615	− 0.481844821	0.018533389
THAP11	− 0.807250427	0.04032733	− 0.593943089	0.018861969
PTPRE	1.565506182	0.000241063	0.504255599	0.019290598
IL11RA	− 0.897536126	0.039623934	− 0.617587742	0.019315582
NARF	0.746465067	0.011958684	0.531794282	0.019361642
TMEM260	− 0.9858146	0.004981655	− 0.472649113	0.019517865
WDR89	0.78862198	0.017657719	− 0.536328947	0.019700691
VAMP3	0.895596534	0.040531984	0.742719501	0.019795093
NVL	− 1.329520343	0.020108611	− 0.660575451	0.020862258
IMPA2	1.710057922	0.008863916	0.55874936	0.020875373
TOP2B	− 1.224847791	0.046556803	− 0.69362205	0.021007495
BACH2	− 1.556070717	0.001593919	− 0.694884856	0.021047149
LOC643072	1.336393236	0.01614887	0.504133706	0.021762187
FAM171A1	− 1.297102946	0.021894851	− 0.857544073	0.021837971
LCN2	1.448749929	0.027264599	0.507488907	0.022287246
F10	0.859648855	0.03887727	0.517023862	0.022463119
RYBP	1.160448619	0.000744752	0.545754325	0.022565273
PVRIG	− 1.048927951	0.012237099	− 0.776672502	0.02315433
POLB	2.156199292	0.000161715	0.7079582	0.023329133
TOP2A	1.220573095	0.042212745	0.531989942	0.023745875
ABHD15	− 0.777671518	0.045324266	− 0.589823096	0.024034951
APOL3	− 1.132962428	0.009183684	− 0.730285885	0.024821715
GNPDA1	− 0.719174212	0.019491228	− 0.64401811	0.025225165
GK3P	0.645446255	0.041071202	0.643697479	0.025486345
MAPK14	1.349924696	0.003860436	0.536455945	0.025679675
CD46	1.670254596	0.020225563	0.612875503	0.025683037
NCF2	0.918872631	0.017642988	0.914126022	0.02604898
CD96	− 1.351341746	0.013543811	− 0.710127733	0.026235883
SLC12A6	1.234381987	0.002745557	0.547304178	0.026259281
LINC00667	− 0.665792667	0.023155853	− 0.564815214	0.026378969
ESYT1	− 1.058244984	0.001054047	− 0.826953028	0.026447069
HMGN3	− 2.10599538	0.01553089	− 0.497207909	0.026987572
POMT1	− 1.167869966	0.004599769	− 0.520023047	0.026988368
TP53TG1	− 0.569201302	0.031094948	− 0.596272312	0.02704165
MTX2	− 1.204498744	0.042747776	− 0.642851561	0.02728884
GPR89B	− 0.869944564	0.048485421	− 0.840069901	0.028144016
PELI2	1.87892659	0.001987193	0.564705661	0.028197457
ZC3H15	1.44627689	0.019429305	0.50345949	0.028393971
RALB	1.752718938	0.003130775	0.575320431	0.028530086
LOC101928615	1.332017065	0.016263326	0.494459535	0.028645539
TUBB2A	4.251553993	0.012679991	1.199901102	0.028677098
ZNF248	− 1.034241515	0.002317027	− 0.484456113	0.028925632
TLR8	1.879547329	0.008044138	0.740129485	0.028974804
STEAP4	2.827840977	0.000560671	0.787317104	0.029413926
ZBTB26	− 0.901579621	0.017961829	− 0.501560605	0.029565582
LINC00847	− 0.922866306	0.006809119	− 0.641852074	0.029566356
TCEAL1	− 1.2531277	0.007061712	− 0.596036376	0.02970108
HBM	5.520539519	0.023178652	0.550713859	0.029743279
POC5	− 1.203888482	0.007930781	− 0.658986077	0.030003161
SRSF4	1.114798217	0.030264809	0.528463588	0.030559463
SMAGP	− 0.654383803	0.009936511	− 0.68678699	0.03089042
MEGF9	2.144879987	0.007476155	0.608436445	0.032247264
CHP1	1.322302286	0.012852758	0.652948587	0.032468912
BIRC6	− 2.268727559	0.019075015	− 0.556815765	0.03283538
STX3	1.667825653	0.006010718	0.466255977	0.033357788
MIR3682	− 1.570178098	0.004819709	− 0.711241423	0.033548018
COTL1	0.98035553	0.005219866	0.498692271	0.034655618
CAMTA2	− 0.682771154	0.02493448	− 0.577222959	0.034861364
IFFO2	− 0.636343197	0.041034444	− 0.530941091	0.03495195
MSANTD2	− 2.400657198	0.014678726	− 0.731603673	0.034988311
MCOLN1	1.947875812	0.040982688	0.474949343	0.035736681
LIMK2	1.16830781	0.025026897	0.485791592	0.035797975
PIK3C2B	− 1.027026319	0.027220986	− 0.714521503	0.036380145
ZSCAN22	− 0.666311572	0.039577441	− 0.628566828	0.036444868
CASP6	− 0.863230967	0.019036775	− 0.452046604	0.036539336
TSEN34	1.100210028	0.014496871	0.502990424	0.036792225
SPIN2B	− 1.080530337	0.00665453	− 0.666560137	0.03687372
DIEXF	− 0.955502636	0.010542312	− 0.480916674	0.036910908
ZNF662	− 1.605391952	0.034739974	− 0.844003226	0.037112399
RLIM	1.517247205	0.002957978	0.48931493	0.037635609
LINC00685	− 0.865718364	0.002554534	− 0.78199725	0.037931288
TFDP1	1.382812362	0.026211996	0.463299209	0.038166365
CKS1B	− 1.260935738	0.035055341	− 0.639890721	0.038265438
MGC27345	− 0.929470759	0.001570377	− 0.765467099	0.038433223
FRG1KP	− 1.483977266	0.005642658	− 0.605037114	0.038906686
CD8A	− 1.035660576	0.011469096	− 0.907658416	0.039051501
LOC284023	− 1.398345512	0.020982512	− 0.659518005	0.039504033
RAB5A	1.022626328	0.002430413	0.457608665	0.039950411
ZNF253	− 0.913362956	0.016033415	− 0.47703892	0.040028712
LOC101929774	− 0.550046909	0.027706997	− 0.524598901	0.040718581
SIAH2	1.502345693	0.044823317	0.681313021	0.040969657
ATP7A	− 0.918566186	0.002800564	− 0.450035247	0.041283805
LRRC69	− 1.351928679	0.001824204	− 0.621229266	0.04146212
FLOT2	1.971830771	0.002734479	0.465792921	0.041477634
ZC3HC1	− 0.612226203	0.013427208	− 0.509214415	0.043063035
SNAP47	− 0.499297152	0.022945868	− 0.530827895	0.043372532
LOC101060391	− 0.684904432	0.010202625	− 0.993794933	0.04414896
CSNK1D	1.001221274	0.042629925	0.508848388	0.04469371
CBX4	1.30318413	0.025436191	0.477334295	0.044771824
LIN7A	1.532754726	0.000495311	0.455799985	0.04570228
AACS	− 0.731283656	0.020694677	− 0.504672718	0.045803274
NIFK-AS1	− 0.80617843	0.036522441	− 0.497446821	0.046488677
LOC100996809	2.815826722	0.0000284	0.475058618	0.047552317
SRGN	1.650008958	0.016200667	0.868334163	0.047684837
ZNF512	− 0.886897601	0.006033191	− 0.656172686	0.047861886
CUZD1	− 1.241274988	0.01051508	− 0.611980841	0.048008528
RPUSD4	− 1.130819146	0.003519244	− 0.475668046	0.048040194
POMP	1.852833565	0.0000716	0.461851078	0.048771334
PDCL3P4	− 1.187208867	0.031281982	− 0.505102304	0.048829835
FAM216A	− 1.516187864	0.019071979	− 0.51801104	0.049031871
C11orf98	− 0.841690629	0.007367045	− 0.618307314	0.049103832
CD160	− 1.475362673	0.02228208	− 0.73506652	0.04928663
PPTC7	1.06135742	0.042924534	0.537282644	0.049610902
PSMC5	− 1.054274949	0.022317354	− 0.67624194	0.049705735

### Functional enrichment analysis and biological network analysis of the conserved genes

To study the biological function of the 477 conserved genes identified, GO enrichment and KEGG pathway analysis were performed. The GO enrichment analysis revealed 211 GO biological processes (Table 2). Response to lipopolysaccharide, response to molecule of bacterial origin and immune system process were the most significantly enriched biological processes. In addition, 23 KEGG pathways were identified through analyzing the conserved genes, among which osteoclast differentiation was considered as the most remarkably enriched pathway (Table 2).

**Table 2:** The top 50 significant GO biological processes and all KEGG pathways enriched by the conserved genes

	*P* value	Term
*GOBPID*		
GO:0032496	< 0.0001	Response to lipopolysaccharide
GO:0002237	< 0.0001	Response to molecule of bacterial origin
GO:0002376	< 0.0001	Immune system process
GO:0006954	< 0.0001	Inflammatory response
GO:0006950	< 0.0001	Response to stress
GO:0009617	< 0.0001	Response to bacterium
GO:0033993	< 0.0001	Response to lipid
GO:0043207	< 0.0001	Response to external biotic stimulus
GO:0051707	< 0.0001	Response to other organism
GO:0006952	< 0.0001	Defense response
GO:0006955	< 0.0001	Immune response
GO:0009607	< 0.0001	Response to biotic stimulus
GO:0009605	< 0.0001	Response to external stimulus
GO:1901700	< 0.0001	Response to oxygen-containing compound
GO:0002526	< 0.0001	Acute inflammatory response
GO:0050900	< 0.0001	Leukocyte migration
GO:0002682	< 0.0001	Regulation of immune system process
GO:0008219	< 0.0001	Cell death
GO:0016265	< 0.0001	Death
GO:0030595	< 0.0001	Leukocyte chemotaxis
GO:0072606	< 0.0001	Interleukin-8 secretion
GO:0001775	< 0.0001	Cell activation
GO:0034097	< 0.0001	Response to cytokine
GO:0071222	< 0.0001	Cellular response to lipopolysaccharide
GO:0019322	< 0.0001	Pentose biosynthetic process
GO:0012501	< 0.0001	Programmed cell death
GO:0050776	< 0.0001	Regulation of immune response
GO:0071219	< 0.0001	Cellular response to molecule of bacterial origin
GO:0006915	< 0.0001	Apoptotic process
GO:0030593	0.000127	Neutrophil chemotaxis
GO:0051704	0.000142	Multi-organism process
GO:0002523	0.000149	Leukocyte migration involved in inflammatory response
GO:0002253	0.000163	Activation of immune response
GO:1990266	0.000178	Neutrophil migration
GO:0002521	0.000181	Leukocyte differentiation
GO:0097530	0.000208	Granulocyte migration
GO:2001242	0.00022	Regulation of intrinsic apoptotic signaling pathway
GO:0045321	0.000232	Leukocyte activation
GO:0060326	0.000258	Cell chemotaxis
GO:0009048	0.000275	Dosage compensation by inactivation of X chromosome
GO:0034201	0.000275	Response to oleic acid
GO:0071216	0.000302	Cellular response to biotic stimulus
GO:0010033	0.000317	Response to organic substance
GO:2001243	0.000351	Negative regulation of intrinsic apoptotic signaling pathway
GO:0032637	0.000409	Interleukin-8 production
GO:0006796	0.000414	Phosphate-containing compound metabolic process
GO:0019362	0.000457	Pyridine nucleotide metabolic process
GO:0046496	0.000457	Nicotinamide nucleotide metabolic process
GO:0033554	0.000588	Cellular response to stress
GO:0070488	0.000596	Neutrophil aggregation
*KEGG-ID*		
4380	< 0.0001	Osteoclast differentiation
5150	< 0.0001	*Staphylococcus aureus* infection
5140	< 0.0001	Leishmaniasis
4610	0.000291	Complement and coagulation cascades
4145	0.000318	Phagosome
4640	0.001751	Hematopoietic cell lineage
5340	0.00454	Primary immunodeficiency
5144	0.005142	Malaria
5145	0.008804	Toxoplasmosis
4670	0.010961	Leukocyte transendothelial migration
5120	0.020107	Epithelial cell signaling in Helicobacter pylori infection
4130	0.026082	SNARE interactions in vesicular transport
910	0.034802	Nitrogen metabolism
5131	0.043143	Shigellosis
4962	0.049527	Vasopressin-regulated water reabsorption
5146	0.050413	Amoebiasis
30	0.052454	Pentose phosphate pathway
4650	0.066291	Natural killer cell mediated cytotoxicity
4060	0.076786	Cytokine–cytokine receptor interaction
4666	0.076955	Fc gamma R-mediated phagocytosis
3410	0.0854	Base excision repair
5014	0.086122	Amyotrophic lateral sclerosis (ALS)
4370	0.092374	VEGF signaling pathway

**Fig. 3:**
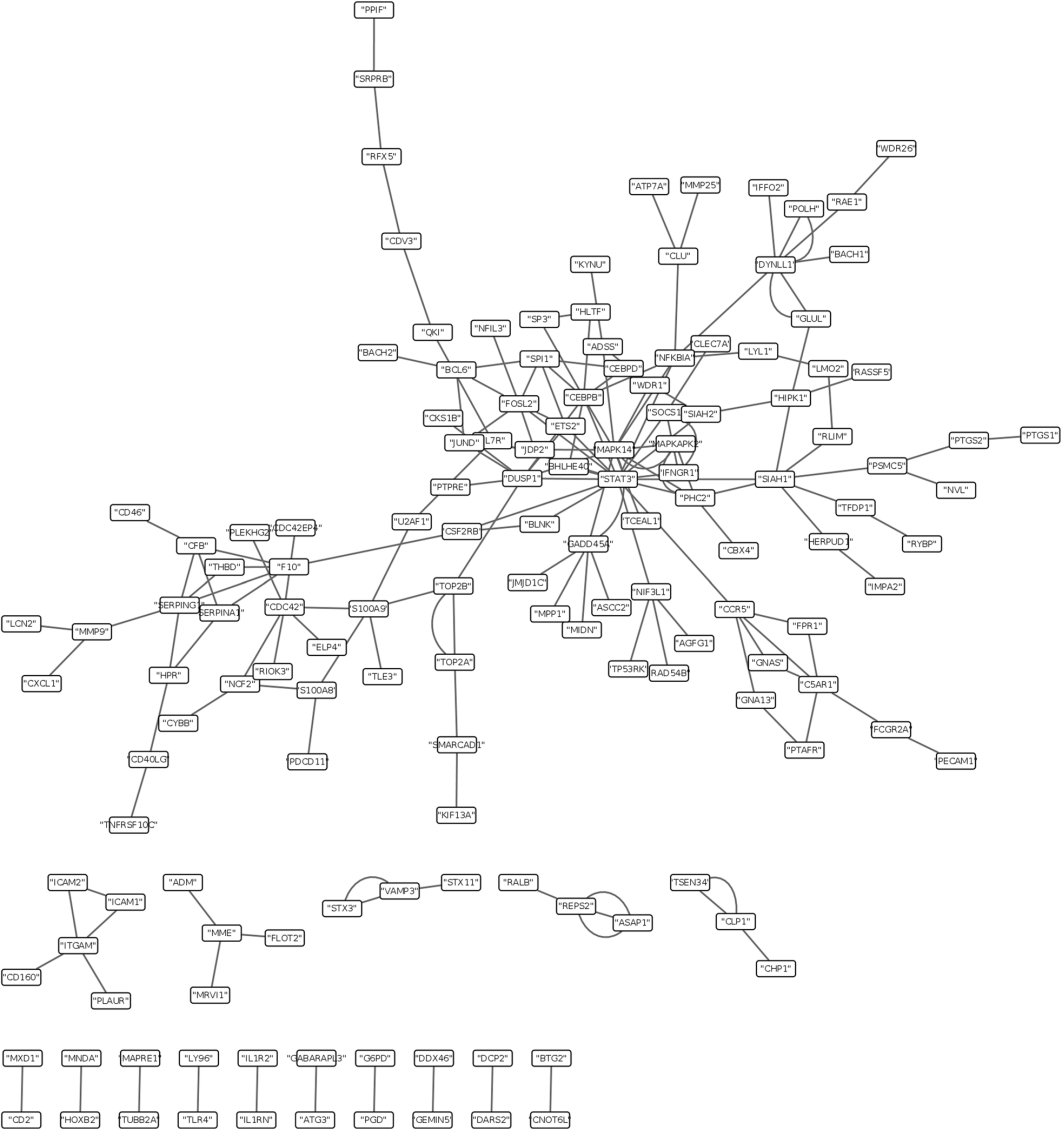
PPI biological network of the conserved genes

To investigate the interaction between the proteins encoded by the conserved genes, protein–protein interaction (PPI) network was employed (Fig. 3). Then, further analysis of critical modules by Cytocluster was carried out. 16 key genes such as *MAPK14*, *STAT3*, and *MAPKAPK2*, were found according to the frequency of genes in critical modules their regulation, which was as follow.

**Table:** 

Genes	GSE97320 (LogFC)	GSE66360 (LogFC)
MAPK14	1.349924696	0.536455945
STAT3	1.780855138	1.024539916
MAPKAPK2	0.765865504	0.778311524

### Validation of the conserved genes using dataset GSE48060

GSE48060 dataset included gene expression profiles of the incident and recurrent MI. Comparison between incident MI and normal control (Comparison 1) revealed 89 DEGs, whereas comparison between recurrent MI and normal control (Comparison 2) showed 392 DEGs (Additional file 1: Table S1 and Table 2). To validate the conserved genes, we overlapped the DEGs of the incident and recurrent MI in GSE48060 and the 477 conserved genes gotten in Comparison 1 and Comparison 2. A total of 29 conserved genes was identified in the overlapping analysis.

### Identification of the potential genes related to recurrent MI

To study the differences between primary and recurrent events of MI on gene expression profiling, we overlapped the DEGs in the incident and recurrent MI. In incident MI, 58 specific DEGs were identified (Table 3), accounting for 65% of the whole DEGs. And they were mainly enriched in 104 GO biological processes and 8 KEGG pathways (Additional file 1: Table S1). In recurrent MI, 361 specific genes were identified (Table 4) as 93% of the whole DEGs, and the functional enrichment analysis revealed 108 GO processes and 21 KEGG pathways (Additional file 2: Table S2). We further overlapped the specific genes in recurrent MI and conserved genes and found that *RNASE2* and *A2M*-*AS1* were potential genes associated with MI recurrence, the regulation of *RNASE2* and *A2M*-*AS1* were 0.629609108 and − 0.936691259.

**Table 3:** Specific DEGs in incident MI

Specific genes	LogFC	*P* value	Specific genes	LogFC	*P* value
LOC400499	0.582246	< 0.0001	ACSL1	0.596533	0.004943
GLT1D1	0.516067	0.000152	INSC	0.461016	0.005675
IL4R	0.53354	0.000156	VNN1	0.579557	0.005783
S100A12	0.870974	0.000159	FCGR1B	0.543697	0.006441
ADM	0.822549	0.000267	FCGR1CP	0.536577	0.006684
SULT1B1	0.565477	0.000335	KLRC2	− 0.59203	0.006728
S100A9	0.555066	0.000392	CYSTM1	0.483289	0.007051
SLPI	0.727612	0.00048	MGAM	0.632329	0.007444
DYSF	0.495236	0.000517	HCAR3	0.526422	0.007467
AQP9	0.623548	0.00055	FOLR3	0.801133	0.007741
NCF4	0.51435	0.000705	LOC100134822	0.482678	0.009538
CR1	0.512093	0.000721	TDRD9	0.549608	0.010174
ANXA3	0.967626	0.000879	FRG1KP	− 0.48373	0.012315
NFE4	0.567447	0.001066	KLRC3	− 0.54028	0.012488
DGAT2	0.477911	0.001101	SLC22A4	0.476108	0.014228
KCNJ15	0.508052	0.001276	TMEM176A	0.465741	0.014307
TXK	− 0.45008	0.001351	FPR2	0.463943	0.014871
SYTL2	− 0.45803	0.001867	NOG	− 0.54482	0.015882
PLBD1	0.512274	0.002083	BCL2A1	0.450316	0.016164
NFIL3	0.573991	0.002206	LRG1	0.505376	0.016586
LMNB1	0.453457	0.002292	MMP9	0.691968	0.023854
FFAR2	0.477117	0.002728	PROK2	0.476171	0.024835
TMEM45A	− 0.63912	0.002983	IL1R2	0.553946	0.029423
PI3	0.61154	0.003202	HSPC102	0.47054	0.033677
DSC2	0.537984	0.003448	LOC107985971	− 0.46157	0.038263
KLRC4	− 0.78034	0.003525	HP	0.529246	0.039574
KRT23	0.606275	0.003532	PFKFB3	0.456526	0.042279
PYGL	0.476712	0.003718	PF4V1	− 0.59584	0.046297
MCEMP1	0.723459	0.004702	HLA-DQA1	− 0.75073	0.048208

**Table 4:** Specific genes in recurrent MI

Specific genes	LogFC	*P* value	Specific genes	LogFC	*P* value
AW029203	− 0.82709	< 0.0001	AW628665	− 0.60526	0.006587
ZNF217	− 0.50883	< 0.0001	CCDC142	− 0.47917	0.006778
AA833902	− 0.67403	< 0.0001	IKBIP	− 0.47796	0.006847
BE552357	− 0.68364	< 0.0001	AA875908	− 0.58822	0.006867
SNAP23	− 0.47426	< 0.0001	CRIM1	− 0.48264	0.006945
AI220134	− 0.46462	< 0.0001	LOC100289230	− 0.46334	0.006947
AK024584	− 1.46739	< 0.0001	HYMAI	− 0.54139	0.007029
AL832672	− 0.70587	< 0.0001	BE219104	− 0.60986	0.007139
H88923	− 0.72215	< 0.0001	HIST1H2AH	− 0.50372	0.007146
PSMA3-AS1	− 0.4581	< 0.0001	AW298171	− 0.47988	0.007146
AK021987	− 0.78158	< 0.0001	LSR	0.455815	0.007277
AA436887	− 0.60722	< 0.0001	TMEM140	− 0.48446	0.007405
RRM2	− 0.53455	< 0.0001	AW771618	− 0.59027	0.007616
CA776505	− 0.76016	0.000117	BC012936	− 0.66788	0.007725
MIR15A	− 0.47975	0.000118	AK024136	− 0.47999	0.007726
BE178502	− 0.6111	0.000129	AW268884	− 0.52578	0.007825
FASLG	− 0.59841	0.000153	AL038450	− 0.53322	0.007986
AL117426	− 0.76868	0.000157	AW291332	− 0.5005	0.00799
AI492388	− 0.99174	0.00016	BQ707256	− 0.55734	0.008015
FOLR1	0.513119	0.000161	AI467945	− 0.60604	0.008019
INAFM2	− 0.62583	0.000165	RHOBTB1	− 0.91345	0.008033
BC043161	− 0.51524	0.000179	FRMD3	− 0.71032	0.008069
RAB27B	− 0.81299	0.000186	AW205919	− 0.46672	0.008619
AA827683	− 0.71156	0.00021	DHRS9	− 0.61396	0.008722
AW452419	− 0.48801	0.000218	AV700081	− 0.74002	0.008968
AL040360	− 0.48226	0.00024	BF509781	− 0.53265	0.009091
LOC100506748	− 0.49388	0.000248	MAP3K7CL	− 0.80885	0.009137
IGF2BP3	− 0.64067	0.000306	AU158247	− 0.53151	0.009175
LINC00877	− 0.50282	0.00031	TNFSF4	− 0.63779	0.00927
STON2	− 0.63358	0.000318	AL036532	− 0.56547	0.009411
BG430958	− 0.83639	0.000335	BE327727	− 0.60652	0.009559
RGS18	− 0.7158	0.00035	AA654772	− 0.4687	0.009566
AW194689	− 0.63151	0.000356	ALDH1A1	− 0.70634	0.009602
AA765387	− 0.63508	0.000363	AW962458	− 0.51864	0.009626
AU158358	− 0.49717	0.000365	PDGFD	− 0.59536	0.009662
AW973253	− 0.80497	0.000382	ZNF304	− 0.6224	0.010013
AW291535	− 0.96722	0.000409	HIST1H4H	− 0.67892	0.010265
AA776723	− 0.59676	0.000421	TRDV3	− 0.64351	0.010276
BZW2	0.704717	0.000437	AV751094	− 0.48494	0.010407
AI916641	− 0.71437	0.000459	HGD	− 0.46226	0.01067
LOC283357	− 0.54561	0.000465	AF116638	− 0.52628	0.010911
AY143171	− 0.57667	0.000465	BC014363	− 0.46713	0.010949
MOB1B	− 0.53099	0.000485	BG484601	− 0.64083	0.011062
AA828246	− 0.45889	0.000487	GPR141	− 0.50505	0.011171
BM353142	− 0.66779	0.000497	MSANTD3-TMEFF1	− 0.46402	0.011211
BF977829	− 0.47673	0.000504	FOS	− 0.73411	0.011316
AW183782	− 0.50329	0.000508	AW151660	− 0.51468	0.01134
AA651631	− 0.59131	0.000533	AA699809	− 0.66516	0.011346
BF195340	− 0.53431	0.000534	GVINP1	− 0.4683	0.011453
SRM	0.452516	0.000552	GPR18	− 0.53962	0.01167
AV711227	− 0.5561	0.000638	BC026299	− 0.78699	0.011784
LOC102723773	− 0.56061	0.000649	AU144136	− 0.54758	0.011809
KBTBD7	− 0.79432	0.00066	BE671138	− 0.48245	0.012101
BE156563	− 0.68551	0.000683	AU147192	− 0.49455	0.012229
AK055960	− 0.52589	0.000684	AL038704	− 0.5175	0.012754
TMEM64	− 0.48231	0.000705	AI798924	− 0.50327	0.012856
PIGB	− 0.65323	0.000718	PRKAR2B	− 0.7607	0.013068
KBTBD6	− 0.67652	0.000727	AW593931	− 0.49127	0.013375
AF085969	− 0.61923	0.000727	AI079544	− 0.4814	0.013515
AL119491	− 0.63799	0.00073	AK024173	− 0.62026	0.013676
BC042590	− 0.61925	0.000775	AI304862	− 0.51052	0.013942
BF197705	− 0.4912	0.00081	AI057404	− 0.46319	0.013973
ZBTB6	− 0.85337	0.00081	AI064690	− 0.55665	0.013997
AK024255	− 0.54336	0.00085	ADRB2	− 0.4926	0.014048
NEXN	− 0.73403	0.000861	BE220061	− 0.48199	0.014631
AA760878	− 0.82077	0.000868	C15orf54	− 0.74653	0.014783
ASGR1	0.581738	0.000898	BF435861	− 0.55039	0.014852
AI021902	− 0.49119	0.00092	ELOVL7	− 0.81121	0.015003
AW172407	− 0.51868	0.000939	PEAR1	− 0.57749	0.01505
SGPP1	− 0.51387	0.000949	SPIN4	− 0.48455	0.015135
AA811257	− 0.57205	0.001008	AI611074	− 0.56408	0.015147
AV702101	− 0.48998	0.001031	AI476542	− 0.51613	0.015174
BF111108	− 0.70231	0.001035	A2M-AS1	− 0.93669	0.015545
GTF2H2B	− 0.64485	0.001037	BG010493	− 0.47885	0.01559
CXCL5	− 1.02412	0.001044	AW827204	− 0.46635	0.015741
HIST1H2BC	− 0.51441	0.001047	AK023294	− 0.45689	0.016361
U54734	− 0.46017	0.001125	CCL5	− 0.51964	0.016688
AA826176	− 0.57887	0.001145	BM970306	− 0.47049	0.016783
SDPR	− 0.69953	0.001222	AW057520	− 0.58156	0.016862
AL049991	− 0.58107	0.001322	TSPAN2	− 0.45957	0.016874
RPS24	− 0.49707	0.00137	ACER2	− 0.46066	0.017131
AV702197	− 0.57669	0.001377	AL137645	− 0.45136	0.017135
RAB30-AS1	− 0.47169	0.001387	AA913146	− 0.5749	0.017371
CPNE2	0.467106	0.00146	EGF	− 0.76368	0.017476
AA620926	− 0.7846	0.001489	AI825538	− 0.51703	0.017663
ASAP2	− 0.87098	0.001503	AW979276	− 0.54705	0.017942
AF127481	− 0.51671	0.00151	AL080280	− 0.45255	0.018173
AF070620	− 0.51609	0.00154	AI629041	− 0.60848	0.01958
AW850555	− 0.62249	0.001583	AI732568	− 0.46701	0.019706
MIR3671	− 0.52361	0.001602	AW467480	− 0.48435	0.019758
W87425	− 0.519	0.001618	AA811657	− 0.47095	0.019921
BF109370	− 0.52884	0.001678	AU144005	− 0.49268	0.020102
AI683805	− 0.78924	0.001783	R34775	− 0.57856	0.020329
ERV3-1	− 0.54004	0.001817	LOC100190986	− 0.45082	0.020747
TBC1D3P1-DHX40P1	− 0.48586	0.00182	CD226	− 0.57555	0.020884
AF090913	− 0.61747	0.001862	CAV2	− 0.55953	0.021661
HIST1H2BH	− 0.6175	0.001893	BF591288	− 0.4639	0.021914
AI754928	− 0.58332	0.001916	HIST2H2BE	− 0.46159	0.021995
GK5	− 0.57178	0.001968	IFNG	− 0.61849	0.022417
AW975051	− 0.58429	0.002076	BE825318	− 0.4626	0.022446
AA521218	− 0.80608	0.002104	P2RY14	− 0.78234	0.022874
NT5C3A	− 0.53059	0.002114	MAF1	0.510049	0.022962
AW590838	− 0.52841	0.00216	AL110175	− 0.4654	0.023008
AI741292	− 0.58062	0.002237	AW168154	− 0.56436	0.023227
BC031345	− 0.49464	0.002274	CTSE	0.469648	0.0234
AI417117	− 0.53753	0.002321	PLGLB1	− 0.59077	0.024037
NORAD	− 0.4814	0.002324	TSPO2	0.49402	0.024729
ERAP2	− 1.08502	0.002351	AA223929	− 0.65433	0.024869
AA682425	− 0.5226	0.002352	LOC145474	− 0.56172	0.025103
AA504261	− 0.91373	0.002354	AI424825	− 0.70976	0.025214
T90348	− 0.83563	0.002381	FAM81B	− 0.65509	0.025317
AI871160	− 0.51414	0.00242	AU122258	− 0.66755	0.0256
AW976631	− 0.50754	0.002429	AI857429	− 0.453	0.025699
AA743565	− 0.4709	0.002437	GRAMD1C	− 0.72878	0.025817
ZNF367	− 0.64369	0.002443	NNT-AS1	− 0.50961	0.025893
ZNF600	− 0.73719	0.00259	SLC25A43	− 0.53296	0.025991
SIRPB2	− 0.58381	0.002601	AW572853	− 0.48068	0.026024
AF119847	− 0.5253	0.00262	AW665840	− 0.55967	0.026095
GCH1	− 0.53975	0.00269	AF289567	− 0.46901	0.026126
MDM1	− 0.45915	0.002735	MS4A7	− 0.59505	0.026763
CCL4	− 0.67183	0.002817	T71269	− 0.54785	0.026781
ZNF431	− 0.62553	0.002854	BC016339	− 0.46302	0.027158
AF075045	− 0.53928	0.002883	ZNF441	− 0.45995	0.027842
ZNF708	− 0.54477	0.002908	AU158442	− 0.9684	0.028345
MIR29C	− 0.52727	0.002958	AI473707	− 0.50584	0.028936
AI347128	− 0.46152	0.003042	CXCL8	− 0.68981	0.030011
T92908	− 0.5946	0.003062	AL079909	− 0.64334	0.030043
GUCY1A3	− 0.73102	0.003102	BF115786	− 0.55711	0.030362
BC010059	− 0.45595	0.003124	ETFDH	− 0.461	0.030362
AK024838	− 0.54078	0.003226	BF357738	0.515919	0.030919
BI052176	− 0.59043	0.003254	BF477544	− 0.45755	0.030986
AI610347	− 0.61651	0.00328	GUCY1B3	− 0.65979	0.031618
AI732181	− 0.66125	0.003311	VNN3	− 0.67564	0.031669
LOC101928625	− 0.53913	0.003343	TMTC3	− 0.45646	0.031671
AK022170	− 0.59907	0.003389	ZNF566	− 0.54998	0.031753
CLIC3	− 0.75131	0.003461	HIST1H2AE	− 0.79566	0.032327
MIR181A2HG	− 0.5147	0.003535	AA521018	− 0.45092	0.033056
AI022132	− 0.50726	0.003542	HCG11	− 0.63207	0.033223
FPGT	− 0.478	0.003602	AW051321	− 0.58	0.033467
TUBB1	− 0.85238	0.003609	AW973834	− 0.47027	0.033714
AK026914	− 0.52808	0.003641	AA916568	− 0.50997	0.033957
BG026159	− 0.45174	0.003642	SGK1	− 0.5908	0.034053
YES1	− 0.4561	0.003815	AI610684	− 0.4633	0.03406
LOC285812	− 0.47746	0.003931	BF115851	− 0.47892	0.034135
TRG-AS1	− 0.51231	0.003988	LOC100653057	0.623258	0.034365
BF062155	− 0.49203	0.004051	BE044089	− 0.49336	0.034417
AI806045	− 0.61316	0.004131	RAD23A	0.548639	0.034488
AL035992	− 0.56231	0.004146	AI703450	− 0.51093	0.034928
W04694	− 0.51849	0.004205	RBM38	0.734977	0.034931
LOC100996741	− 0.49958	0.004209	AW297731	− 0.73551	0.035363
PPBP	− 0.57347	0.004344	AI806781	− 0.60057	0.035437
HTRA1	0.455108	0.004386	MAL	0.546236	0.035497
H57111	− 0.51543	0.004428	AW418562	− 0.51123	0.036077
PMAIP1	− 0.64317	0.004506	SPARC	− 0.70474	0.036622
AU146310	− 0.47187	0.00458	AA250831	− 0.58747	0.036742
AI921882	− 0.52074	0.004596	FLVCR1	− 0.47054	0.037183
CA442689	− 0.66622	0.004599	TRBV27	− 0.86769	0.037188
GOLGA8 N	− 0.46539	0.004601	AW664903	− 0.49026	0.037975
BC020933	− 0.46219	0.004688	BC022885	− 0.60357	0.038476
PGRMC1	− 0.58512	0.004819	BE467916	− 0.54343	0.039249
MINOS1P1	− 0.65789	0.004836	BC006164	− 1.20815	0.040038
AW137073	− 0.54917	0.004961	AW270499	− 0.49699	0.042642
T97544	− 0.61727	0.00501	AU144781	− 0.49058	0.0429
AI308174	− 0.4601	0.005026	SPAG1	− 0.48574	0.043545
BC034024	− 0.45108	0.005106	C2orf88	− 0.48775	0.043772
HIST1H2AC	− 0.5108	0.005249	BF115815	− 0.59141	0.043851
R71414	− 0.66163	0.005499	LOC101060391	0.460163	0.045026
AU155384	− 0.70942	0.005643	GP6	− 0.58096	0.045081
CCL3L3	− 0.80038	0.005666	AI524996	− 0.5128	0.045898
LOC100996756	0.561801	0.005688	BC033945	− 0.48403	0.045936
GTF2H2C_2	− 0.50582	0.005701	PTGS2	− 0.84183	0.04654
PTGDR	− 0.51982	0.005712	RNASE2	0.629609	0.046565
H55978	− 0.48956	0.005729	ATL1	− 0.46269	0.046657
AI700476	− 0.64994	0.005945	FNBP1L	− 0.46579	0.046999
MEIS1	− 0.71932	0.006128	AI743261	− 0.46303	0.047672
BC036606	− 0.74748	0.006153	BQ446762	− 0.4953	0.048681
AW850611	− 0.45324	0.006164	BE856980	− 0.45498	0.048921
BF591615	− 0.56006	0.006345	SLC35D3	− 0.76769	0.049055
AW014108	− 0.46355	0.006419	BF725688	− 0.50505	0.049093
ABCA7	0.486407	0.00648	BF224430	− 0.55108	0.04927
PROSER2	− 0.51582	0.006568			

## Discussion

The present study not only identifies conserved genes and dysregulated pathways in MI but also reveals several hub genes, such as *MAPK14*, *STAT3*, and *MAPKAPK2*. Gene expression alterations of the incident and recurrent MI reveals significant differences. *RNASE2* and *A2M*-*AS1* were identified as potential genes associated with MI recurrence. Those genes could serve as potential biomarkers for MI occurrence or recurrence prediction and diagnosis.

MAPK14, also known as p38α, is one of the four p38 MAPKs, including α, β, δ, and ε isoforms and is the most abundant isoform in human cardiac tissue [14, 15]. P38 MAPK was first reported to be activated by ischemia/reperfusion (I/R) injury [16]. During myocardial ischemia, MAPK14 is found to contribute to infarction, and short-term intraischemic inhibition of this MAPK14 in the intact heart reduces infarction [17]. However, the effect of p38 MAPK on MI is controversial. Mitra et al. has demonstrated that p38 MAPK actually decreases ischemic load during MI, and plays a dual role in pro-survival as well as cardioprotective during ischemia in the absence of reperfusion [18]. The presented study showed MAPK14 upregulation in MI compared with normal tissue. MAPKAPK2 (MAPK-activated protein kinase 2) gene encodes a member of the Ser/Thr protein kinase family. This kinase is regulated through direct phosphorylation by p38 MAP kinase [19]. Inhibition of p38 MAPK leads to a significant decrease in the phosphorylation status of MAPKAPK2 [18]. In conjunction with p38 MAP kinase, MAPKAPK2 is known to be involved in many cellular processes including stress and inflammatory responses, nuclear export, gene expression regulation and cell proliferation [19]. Heat shock protein HSP27 was shown to be one of the substrates of MAPKAPK2and MAPKAPK2 phosphorylates Akt in neutrophils [20]. The isolated perfused rat heart reveals that global ischemia activates *MAPKAPK2*, and this activation is maintained during reperfusion [16]. *MAPKAPK2* has been regarded as a biomarker in MI early stage and recovery [4]. STAT3 (Signal transducer and activator of transcription 3) is required for myocardial capillary growth, control of interstitial matrix deposition, and heart protection from ischemic injury [21]. *STAT3* deficiency causes enhanced susceptibility to myocardial ischemia/reperfusion injury and infarction with increased cardiac apoptosis, increased infarct sizes, and reduced cardiac function and survival [21]. In addition, knockout of *STAT3* in mice treated with lipopolysaccharide leads to more cardiac THF production, and fibrosis [22]. Therefore, *MAPK14*, *STAT3*, and *MAPKAPK2* might be regarded as biomarkers in MI, and the other hub genes are also deserved to be further studies.

Compared to incident cases of MI, recurrent cases of MI experienced more often heart failure, impaired left ventricular ejection fraction, and multivessel disease [23]. In this study, the gene expression profiling between first and recurrent MI showed significant differences, evidenced by that 93% of the whole DEGs in recurrent MI were its specific genes. *RNASE2* and *A2M*-*AS1* were regarded as potential genes associated with MI recurrence. RNASE2 gene encodes an enzyme in humans called eosinophil-derived neurotoxin (EDN) [24, 25]. EDN is one of the four major secretory proteins found in the specific granules of the human eosinophilic leukocyte and has been detected in eosinophils, specifically monocytes, and dendritic cells as well as in basophils and neutrophils [26]. EDN was first identified as a neurotoxin, and recent studies suggest that EDN plays a role in antiviral host defense, as a chemoattractant for human dendritic cells, and most recently, as an endogenous ligand for toll-like receptor (TLR) 2 [27]. TLR2 is reported to regulate myocardial ischemia, and sTLR2 may involve in the innate immune response in the pathogenesis of heart failure after acute MI [28]. Thus, we hypothesize that EDN/RNASE2 is likely to be associated with recurrent MI via its direct interactions with TLR2 and dendritic cells. Though little knowledge is available on A2M antisense RNA 1 (A2M-AS1), the relationship between A2M and MI has been reported. The cardiac isoform of A2M (cardiac A2M) is considered as an early marker in cardiac hypertrophy and left ventricular mass in humans. And the further study reveals that cardiac A2M is a valuable marker for the diagnosis of MI diabetic patients and differentiating them from diabetic patients without MI [29]. Thus, the role of *A2M*-*AS1* in recurrent MI need to be further investigated in the future study.

## Conclusions

Lacking animal models and cell culture experiment validation are limitations to our study. As an alternative way of validation, here we used GSE48060 dataset to validate the conserved genes. However, further functional experiments are needed to investigate the role of these candidate genes in myocardial infarction though we have reviewed their related functions reported in the previous publication. Meanwhile, the single-nucleotide polymorphism of these candidates may be associated with the risk of heart disease, also deserving for the future investigation. In addition, though myocardial tissues well reflect the characteristics of the injury areas, the blood samples may facilitate clinical diagnosis and treatment via the target genes in the future.

## Additional files

**Additional file 1: Table S1.** The top 50 significant GO biological processes and all KEGG pathways enriched by the specific genes in incident MI.

**Additional file 2: Table S2.** The top 50 significant GO biological processes and all KEGG pathways enriched by the specific genes in recurrent MI.

## Data Availability

The datasets used and/or analyzed during the current study are available from the corresponding author on reasonable request.

## Abbreviations

MI: myocardial infarction
DEGs: differentially expressed genes
PPI: protein–protein-interaction
PCI: percutaneous coronary intervention
ECG: electrocardiogram
CKMB: MB (muscle/brain) fraction of creatine kinase
GO: gene ontology
KEGG: Kyoto Encyclopedia of Genes and Genomes
GEO: gene expression omnibus
MAPK: mitogen-activated protein kinase
MAPKAPK2: MAPK-activated protein kinase 2
STAT3: signal transducer and activator of transcription 3
EDN: eosinophil-derived neurotoxin
TLR2: toll-like receptor 2
RNASE2: human ribonuclease 2
A2M-AS1: A2M antisense RNA 1
