# Supplementary material for: Identification of key genes involved in myocardial infarction
Source: Eur J Med Res. 2019 Jul 3;24:22. doi: 10.1186/s40001-019-0381-x (PMC6607516; doi:10.1186/s40001-019-0381-x)
Supplement: Supplementary file 1 — Additional file 1: Table S1. The top 50 significant GO biological processes and all KEGG pathways enriched by the specific genes in incident MI. [file 40001_2019_381_MOESM1_ESM.docx]

Table S1 The top 50 significant GO biological processes and all KEGG pathways enriched by the specific genes in incident MI

| GOBPID | P value | Term |
| --- | --- | --- |
| GO:0006952 | <0.0001 | defense response |
| GO:0006955 | <0.0001 | immune response |
| GO:0006954 | <0.0001 | inflammatory response |
| GO:0002376 | <0.0001 | immune system process |
| GO:0006950 | <0.0001 | response to stress |
| GO:0070887 | <0.0001 | cellular response to chemical stimulus |
| GO:0034201 | 0.000131 | response to oleic acid |
| GO:0051240 | 0.000219 | positive regulation of multicellular organismal process |
| GO:0071345 | 0.000249 | cellular response to cytokine stimulus |
| GO:0019221 | 0.000305 | cytokine-mediated signaling pathway |
| GO:0002526 | 0.000543 | acute inflammatory response |
| GO:0002523 | 0.000569 | leukocyte migration involved in inflammatory response |
| GO:0070542 | 0.000729 | response to fatty acid |
| GO:0034097 | 0.000751 | response to cytokine |
| GO:0042221 | 0.000752 | response to chemical |
| GO:0006968 | 0.000804 | cellular defense response |
| GO:0009605 | 0.000842 | response to external stimulus |
| GO:0032602 | 0.000883 | chemokine production |
| GO:2001242 | 0.000897 | regulation of intrinsic apoptotic signaling pathway |
| GO:1900120 | 0.0009 | regulation of receptor binding |
| GO:0006935 | 0.000918 | chemotaxis |
| GO:0042330 | 0.000918 | taxis |
| GO:0045582 | 0.000925 | positive regulation of T cell differentiation |
| GO:0030595 | 0.001178 | leukocyte chemotaxis |
| GO:0051704 | 0.001224 | multi-organism process |
| GO:0045621 | 0.001465 | positive regulation of lymphocyte differentiation |
| GO:0045087 | 0.00152 | innate immune response |
| GO:0002521 | 0.001792 | leukocyte differentiation |
| GO:0002544 | 0.001953 | chronic inflammatory response |
| GO:0035336 | 0.002135 | long-chain fatty-acyl-CoA metabolic process |
| GO:0060333 | 0.002242 | interferon-gamma-mediated signaling pathway |
| GO:0009617 | 0.002401 | response to bacterium |
| GO:2001243 | 0.00247 | negative regulation of intrinsic apoptotic signaling pathway |
| GO:0046903 | 0.002541 | secretion |
| GO:0050729 | 0.002548 | positive regulation of inflammatory response |
| GO:0050900 | 0.002614 | leukocyte migration |
| GO:0030217 | 0.002627 | T cell differentiation |
| GO:0034109 | 0.002906 | homotypic cell-cell adhesion |
| GO:2001234 | 0.002933 | negative regulation of apoptotic signaling pathway |
| GO:0009251 | 0.00294 | glucan catabolic process |
| GO:0071353 | 0.00294 | cellular response to interleukin-4 |
| GO:0002823 | 0.00316 | negative regulation of adaptive immune response based on somatic recombination of immune receptors built from immunoglobulin superfamily domains |
| GO:0044247 | 0.00316 | cellular polysaccharide catabolic process |
| GO:0050832 | 0.00316 | defense response to fungus |
| GO:0071310 | 0.00323 | cellular response to organic substance |
| GO:0045580 | 0.003236 | regulation of T cell differentiation |
| GO:0035337 | 0.003388 | fatty-acyl-CoA metabolic process |
| GO:1900408 | 0.003388 | negative regulation of cellular response to oxidative stress |
| GO:1902883 | 0.003388 | negative regulation of response to oxidative stress |
| GO:0042742 | 0.003438 | defense response to bacterium |
| KEGGID |  |  |
| 4612 | 0.000191 | Antigen processing and presentation |
| 5140 | 0.002629 | Leishmaniasis |
| 4640 | 0.004644 | Hematopoietic cell lineage |
| 4670 | 0.009987 | Leukocyte transendothelial migration |
| 500 | 0.018579 | Starch and sucrose metabolism |
| 5150 | 0.019235 | Staphylococcus aureus infection |
| 4060 | 0.082823 | Cytokine-cytokine receptor interaction |
| 4650 | 0.098313 | Natural killer cell mediated cytotoxicity |
