# Supplementary material for: Identification of key genes involved in myocardial infarction
Source: Eur J Med Res. 2019 Jul 3;24:22. doi: 10.1186/s40001-019-0381-x (PMC6607516; doi:10.1186/s40001-019-0381-x)
Supplement: Supplementary file 2 — Additional file 2: Table S2. The top 50 significant GO biological processes and all KEGG pathways enriched by the specific genes in recurrent MI. [file 40001_2019_381_MOESM2_ESM.docx]

Table S2 The top 50 significant GO biological processes and all KEGG pathways enriched by the specific genes in recurrent MI

| GOBPID | P value | Term |
| --- | --- | --- |
| GO:0032496 | <0.0001 | response to lipopolysaccharide |
| GO:0009607 | <0.0001 | response to biotic stimulus |
| GO:0002237 | <0.0001 | response to molecule of bacterial origin |
| GO:0043207 | <0.0001 | response to external biotic stimulus |
| GO:0051707 | <0.0001 | response to other organism |
| GO:0009617 | <0.0001 | response to bacterium |
| GO:0002690 | <0.0001 | positive regulation of leukocyte chemotaxis |
| GO:0045986 | <0.0001 | negative regulation of smooth muscle contraction |
| GO:0002376 | 0.000131 | immune system process |
| GO:0006925 | 0.000164 | inflammatory cell apoptotic process |
| GO:0002688 | 0.000193 | regulation of leukocyte chemotaxis |
| GO:0006952 | 0.000246 | defense response |
| GO:0045932 | 0.00027 | negative regulation of muscle contraction |
| GO:0002687 | 0.000282 | positive regulation of leukocyte migration |
| GO:0045672 | 0.000362 | positive regulation of osteoclast differentiation |
| GO:0070098 | 0.000396 | chemokine-mediated signaling pathway |
| GO:2000503 | 0.000403 | positive regulation of natural killer cell chemotaxis |
| GO:0006954 | 0.000421 | inflammatory response |
| GO:0060326 | 0.000457 | cell chemotaxis |
| GO:0030595 | 0.000488 | leukocyte chemotaxis |
| GO:0002714 | 0.000533 | positive regulation of B cell mediated immunity |
| GO:0002891 | 0.000533 | positive regulation of immunoglobulin mediated immune response |
| GO:0008217 | 0.00054 | regulation of blood pressure |
| GO:0042127 | 0.000597 | regulation of cell proliferation |
| GO:0050921 | 0.000626 | positive regulation of chemotaxis |
| GO:0032103 | 0.000632 | positive regulation of response to external stimulus |
| GO:0033993 | 0.000669 | response to lipid |
| GO:0030335 | 0.000955 | positive regulation of cell migration |
| GO:0050900 | 0.001057 | leukocyte migration |
| GO:0045744 | 0.001061 | negative regulation of G-protein coupled receptor protein signaling pathway |
| GO:2000147 | 0.0011 | positive regulation of cell motility |
| GO:2000501 | 0.001114 | regulation of natural killer cell chemotaxis |
| GO:0008015 | 0.001142 | blood circulation |
| GO:0003013 | 0.001182 | circulatory system process |
| GO:0002685 | 0.001217 | regulation of leukocyte migration |
| GO:0048520 | 0.001217 | positive regulation of behavior |
| GO:0051272 | 0.001312 | positive regulation of cellular component movement |
| GO:0009629 | 0.001426 | response to gravity |
| GO:0006898 | 0.001495 | receptor-mediated endocytosis |
| GO:0040017 | 0.001526 | positive regulation of locomotion |
| GO:0006897 | 0.001576 | endocytosis |
| GO:0006670 | 0.001775 | sphingosine metabolic process |
| GO:0010561 | 0.001775 | negative regulation of glycoprotein biosynthetic process |
| GO:0035747 | 0.001775 | natural killer cell chemotaxis |
| GO:0002889 | 0.001984 | regulation of immunoglobulin mediated immune response |
| GO:0002712 | 0.002135 | regulation of B cell mediated immunity |
| GO:0050920 | 0.002144 | regulation of chemotaxis |
| GO:0007171 | 0.00216 | activation of transmembrane receptor protein tyrosine kinase activity |
| GO:0032700 | 0.00216 | negative regulation of interleukin-17 production |
| GO:0034311 | 0.00216 | diol metabolic process |
| KEGGID |  |  |
| 5322 | 2.49E-05 | Systemic lupus erythematosus |
| 4060 | 0.000104 | Cytokine-cytokine receptor interaction |
| 5323 | 0.00015 | Rheumatoid arthritis |
| 5142 | 0.000313 | Chagas disease (American trypanosomiasis) |
| 4540 | 0.001219 | Gap junction |
| 4062 | 0.006718 | Chemokine signaling pathway |
| 4620 | 0.013126 | Toll-like receptor signaling pathway |
| 5140 | 0.026778 | Leishmaniasis |
| 4144 | 0.03402 | Endocytosis |
| 5143 | 0.039379 | African trypanosomiasis |
| 5330 | 0.043579 | Allograft rejection |
| 4970 | 0.045849 | Salivary secretion |
| 600 | 0.050176 | Sphingolipid metabolism |
| 5332 | 0.052451 | Graft-versus-host disease |
| 5219 | 0.054762 | Bladder cancer |
| 4940 | 0.057109 | Type I diabetes mellitus |
| 230 | 0.05763 | Purine metabolism |
| 480 | 0.074469 | Glutathione metabolism |
| 5144 | 0.077073 | Malaria |
| 4623 | 0.090511 | Cytosolic DNA-sensing pathway |
| 4621 | 0.096068 | NOD-like receptor signaling pathway |
